# *LINC01232* serves as a novel biomarker and promotes tumour progression by sponging miR-204-5p and upregulating RAB22A in clear cell renal cell carcinoma

**DOI:** 10.1080/07853890.2021.2001563

**Published:** 2021-11-16

**Authors:** Qingling Liu, Chengbin Lei

**Affiliations:** a Department of Clinical Laboratory, Zibo Maternal and Child Health Hospital, Shandong, China; b Department of Clinical Laboratory, Zibo Central Hospital, Shandong, China

**Keywords:** *LINC01232*, miR-204-5p, RAB22A, clear cell renal cell carcinoma, prognosis

## Abstract

**Background:**

Long non-coding RNAs (lncRNAs) are involved in the progression of various cancers, including clear cell renal cell carcinoma (ccRCC). This study aimed to investigate the expression and prognostic value of long intergenic non-protein coding RNA (LINC) 01232 in ccRCC and preliminary explore the molecular mechanism underlying the role of *LINC01232* in ccRCC progression.

**Methods:**

Tumour tissues and adjacent normal tissues of 122 patients with ccRCC were collected in this study. The levels of *LINC01232*, microRNA (miR)-204-5p and RAB22A were measured by quantitative real-time PCR. The proliferation, migration and invasion of ccRCC cells were detected by cell counting kit-8 assay and Transwell assay, respectively. The interaction among *LINC01232*, miR-204-5p and RAB22A was confirmed by bioinformatics analysis, dual-luciferase reporter assay and Pearson correlation analysis. The association of *LINC01232* and miR-204-5p with ccRCC patient survival was verified by the Kaplan–Meier method and log-rank test. The prognostic value of *LINC01232* in ccRCC was confirmed by Cox regression analysis.

**Results:**

*LINC01232* expression was increased in ccRCC tumour tissues and ccRCC cells and independently predicted the prognosis of ccRCC patients. In addition, *LINC01232* silencing inhibited ccRCC cell proliferation, migration and invasion. Moreover, *LINC01232* served as a sponge for miR-204-5p, and miR-204-5p reduction reversed the inhibitory effect of *LINC01232* silencing on ccRCC cell function. Furthermore, *LINC01232* could sponge miR-204-5p, causing the elevation of RAB22A in ccRCC, thereby promoting ccRCC cell function.

**Conclusion:**

*LINC01232* may be an independent prognostic biomarker in ccRCC and plays an oncogenic role in ccRCC progression by sponging miR-204-5p and upregulating RAB22A.

## Introduction

Renal cell carcinoma (RCC), a cancer that occurs in renal epithelial cells, is one of the most common and lethal malignancies [1]. The most common subtype of RCC is clear cell RCC (ccRCC), which accounts for approximately 80% of adult clinical RCC cases [2]. Localized ccRCC can be treated by partial or total resection, but most patients are initially diagnosed at an advanced stage, and advanced ccRCC remains a clinical challenge with a 5-year overall survival rate of less than 20% and a poor prognosis [3,4]. Thus, there is an urgent need to explore reliable prognostic biomarkers and novel approaches to improve the prognosis and treatment of patients with ccRCC.

Long non-coding RNAs (lncRNAs) are RNAs greater than 200 nucleotides in length that cannot encode proteins [5]. At present, the roles of lncRNAs in malignant tumours have been gradually unravelled, providing some new potential targets for the treatment of diseases [6–8]. However, there have been fewer functional lncRNA analyses in ccRCC. Our previous studies have demonstrated that long intergenic non-protein coding RNA (LINC) 01232 has important biological functions and clinical significance in pancreatic adenocarcinoma (PAAD) [9,10]. In addition, Zhao et al. have reported that *LINC01232* is highly expressed in oesophageal squamous cell carcinoma (ESCC) tissues and ESCC cell lines and can regulate the biological functions of ESCC cells [11]. Moreover, similar results were reported regarding *LINC01232* in oral carcinoma (OC) [12]. In this study, by using a bioinformatics analysis platform, we found that the expression levels of *LINC01232* in ccRCC tumour tissues were significantly upregulated and significantly correlated with disease prognosis. However, until now, there has been no report on *LINC01232* in ccRCC.

Therefore, the purpose of this study was to analyse the expression level of *LINC01232* in ccRCC tumour tissues and ccRCC cell lines, explore the role of *LINC01232* in ccRCC tumour progression using *in vitro* experiments, and provide a preliminary exploration of its molecular mechanisms. This study may provide a new and effective biomarker for prognosis prediction of ccRCC patients and a potential target for ccRCC therapy.

## Material and methods

### Patients and tissue collection

Tissue samples, including tumour tissues and adjacent normal tissues (at least 5 cm away from the tumour), were collected from 122 ccRCC patients who underwent surgical resection at Zibo Maternal and Child Health Hospital from 2010 to 2016. None of the patients had received radiotherapy or chemotherapy before surgery, and all patients were diagnosed with ccRCC by pathological examination. All tissues were collected during surgery and immediately stored in liquid nitrogen until use. Each patient has signed informed consent and the procedures for all experiments were approved by the Ethics Committee of Zibo Maternal and Child Health Hospital. All patients were followed up for five years, and survival information was recorded.

### Cell culture and transfection

A human normal renal tubular epithelial cell line HK-2 and five ccRCC cell lines (Caki-1, A498, KN-41, 786-O and ClearCa-1) were purchased from the Cell Bank of Chinese Academy of Sciences (Shanghai, China). The cells were cultured in Dulbecco’s Modified Eagle’s Medium (DMEM, Invitrogen, Thermo Fisher Scientific, Waltham, MA) containing 10% foetal bovine serum (FBS, Invitrogen), 100 U/mL penicillin and 100 μg/mL streptomycin.

The short hairpin RNA (shRNA) against *LINC01232* (sh-*LINC01232*), the corresponding negative control (sh-NC), microRNA (miR)-204-5p mimic, mimic NC, miR-204-5p inhibitor and inhibitor NC were synthesized by GenePharma. The sequences were as follows: sh-*LINC01232*, CCGCGACACGTCATCTAGAATAACTCHAGTTATTCTAGATGACGTGTCTTTTTG, and sh-NC, CCGCGGACTTGCCTCCTACACTACTCHAGTAGTGTAGGAGGCAAGTCCTTTTTG; miR-204-5p mimic, 5′-UUCCCUUUGUCAUCCUAUGCCU-3′, and mimic NC, 5′-UUCUCCGAACGUGUCACGU-3′; miR-204-5p inhibitor, 5′-AGGCAUAGGAUGACAAAGGGAA-3′, and inhibitor NC, 5′-CAGUACUUUUGUGUAGUACAA-3′. The above fragments were respectively transfected into Caki-1 and 786-O cells using Lipofectamine 3000 (Invitrogen, USA). The pcDNA3.1 and pcDNA3.1-*LINC01232* expression vectors were synthesized by GenePharma and were transfected into Caki-1 cells by Lipofectamine 3000 (Invitrogen, USA).

### Bioinformatics analysis

In this study, the expression level of *LINC01232* and its relationship with ccRCC patient survival in TCGA database were analysed using starBase v3.0 [13] platform (http://starbase.sysu.edu.cn/index.php), and the combination of miR-204-5p with *LINC01232* was predicted by this platform. In addition, by using the starBase v3.0 platform, we analysed the expression level of miR-204-5p and its correlation with survival of ccRCC patients in TCGA database, and further analysed the correlation among *LINC01232*, miR-204-5p and RAB22A in ccRCC patients. Moreover, starBase v3.0 platform was used to analyse the relationship of RAB22A with ccRCC patient survival in TCGA database.

### RNA extraction and quantitative real-time PCR (qRT-PCR)

Total RNA was extracted using the TRIzol reagent (Invitrogen, Carlsbad, CA). A NanoDrop 2000 (Thermo Fisher Scientific, Waltham, MA) was utilized to verify RNA purity and concentration. Then, a Reverse Transcription kit (Thermo Fisher Scientific, Waltham, MA) was used for reverse transcription to synthesize the cDNA. The qRT-PCR was carried out using a 7500 Real-Time PCR System (Applied Biosystems, USA) with SYBR green I Master Mix kit (Invitrogen, Carlsbad, CA) for the detection of *LINC01232*, RAB22A and miR-204-5p levels. The levels of *LINC01232* and RAB22A were normalized to GAPDH, and miR-204-5p levels were normalized to U6. Their levels were calculated using the 2^−ΔΔCt^ method [14].

### Cell proliferation analysis

Proliferation of Caki-1 and 786-O cells was assessed by cell counting kit-8 (CCK-8) assay. The ccRCC cells were seeded in 96-well cell culture plates at a cell density of 3 × 10^3^ cells/well. After incubation at 37 °C for 24, 48 and 72 h, 10 µl CCK-8 reagent was added to each well. Cells were then placed in an incubator at 37 °C for another 2 h. Then, the optical density (OD) value at 450 nm was detected using a microplate analyser (Bio-Rad Laboratories, Inc.).

### Cell migration and invasion analysis

The migration and invasion abilities of Caki-1 and 786-O cells were measured using Transwell assays. For invasion assays, Transwell chambers were precoated with Matrigel (Corning, USA), and Transwell chambers without Matrigel precoating were used for migration assays. For each analysis, cells (5 × 10^4^) were added to the upper chamber with serum-free medium; meanwhile, the lower chamber was filled with serum medium. After 24-h incubation at 37 °C, cells on the upper chamber membrane were wiped away. Cells on the bottom side were then fixed with 4% paraformaldehyde and stained with 0.1% crystal violet for 20 min at room temperature. The number of cells was counted under an inverted light microscope (Olympus Corporation, Tokyo, Japan).

### Dual-luciferase reporter assay

The wild-type (WT)-*LINC01232*, mutant-type (MUT)-*LINC01232*, WT-RAB22A and MUT-RAB22A were inserted into the pmirGLO dual luciferase reporter vector (Promega, WI). The WT or MUT vector was then co transfected with miR-204-5p mimic or mimic NC into Caki-1 cells using Lipofectamine 3000 reagent (Invitrogen, Carlsbad, CA). After 48 h, the luciferase activity was measured using the dual-luciferase reporter assay system (Promega). Firefly luciferase activity was normalized to *Renilla* luciferase activity. All procedures followed the manufacturer’s instructions.

### Statistical analysis

Data analysis results were expressed as mean ± SD. All analyses were performed by SPSS 22.0 (IBM Corp.) and GraphPad Prism 7.0 software (GraphPad Software, Inc.), and repeated independently at least three times. Differences in measurement data between two groups and among multiple groups were analysed by using Student’s *t*-test and one-way ANOVA followed by Tukey’s post hoc test, respectively. Comparison between categorical variables was conducted by Chi-square test. Pearson correlation analysis was performed to analyse the correlation between two variables (including *LINC01232*, RAB22A and miR-204-5p). Kaplan–Meier survival curves and log-rank tests were utilised to investigate the relationship of *LINC01232* and miR-204-5p expression with overall survival of ccRCC patients. Cox regression analysis was used to identify the prognostic value of *LINC01232* in ccRCC patients. *p* < .05 indicated a statistically significant difference.

## Results

### Overexpression of LINC01232 in ccRCC tissues and cell lines

**Figure 1.**
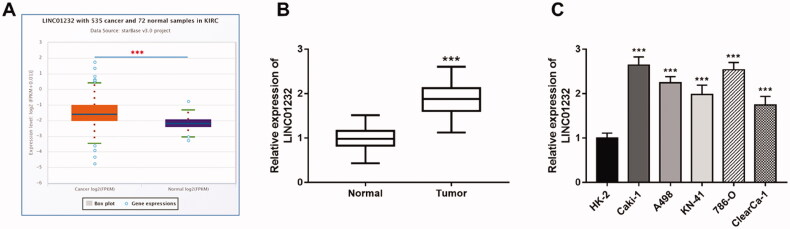
Overexpression of *LINC01232* in ccRCC tissues and cell lines. (A) *LINC01232* expression in 535 ccRCC tissues and 72 normal tissues from TCGA database by starBase v3.0 analysis. (B) *LINC01232* expression in tumour tissues and normal tissues from 122 ccRCC patients from our study cohort (fold change is 0.89). (C) *LINC01232* expression in ccRCC cell lines and normal renal tubular epithelial cell line (fold changes are 2.64, 2.25, 1.98, 2.54 and 1.75, respectively). ***p < .001 vs. ccRCC tissues from TCGA database or normal tissues from 122 ccRCC patients or HK-2. LINC: long intergenic non-protein coding RNA; ccRCC: clear cell renal cell carcinoma.

The expression data of 535 ccRCC tissues and 72 normal tissues from TCGA database were analysed in starBase v3.0 platform, and the results indicated that *LINC01232* was significantly upregulated in ccRCC tissues compared with that in normal tissues (Figure 1(A), *p*<.001). The expression of *LINC01232* in other RCC subtypes from TCGA dataset was analysed in starBase v3.0 platform and presented in Figure S1. *LINC01232* expression in kidney chromophobe (KICH) was decreased (Figure S1(a), *p* < .001), and *LINC01232* expression in kidney renal papillary cell carcinoma (KIRP) was increased (Figure S1(b), *p* < .001). In addition, the expression of *LINC01232* in other types of cancer was also assessed using the TCGA dataset (Figure S2). In addition to ccRCC and KIRP, the significantly increased *LINC01232* expression was also found in colon adenocarcinoma (COAD; Figure S2(a), *p* < .001), oesophageal carcinoma (ESCA; Figure S2(b), *p* < .01), lung squamous cell carcinoma (LUSC; Figure S2(c), *p* < .001) and prostate adenocarcinoma (PRAD; Figure S2(d), *p* < .001). Consistent with this result, tumour tissues from 122 ccRCC patients in this study had significantly higher *LINC01232* levels than adjacent normal tissues (Figure 1(B), *p*<.001), and *LINC01232* expression was similarly increased in ccRCC cell lines compared with that in normal cells (Figure 1(C), all *p* < .001).

**Figure 2.**
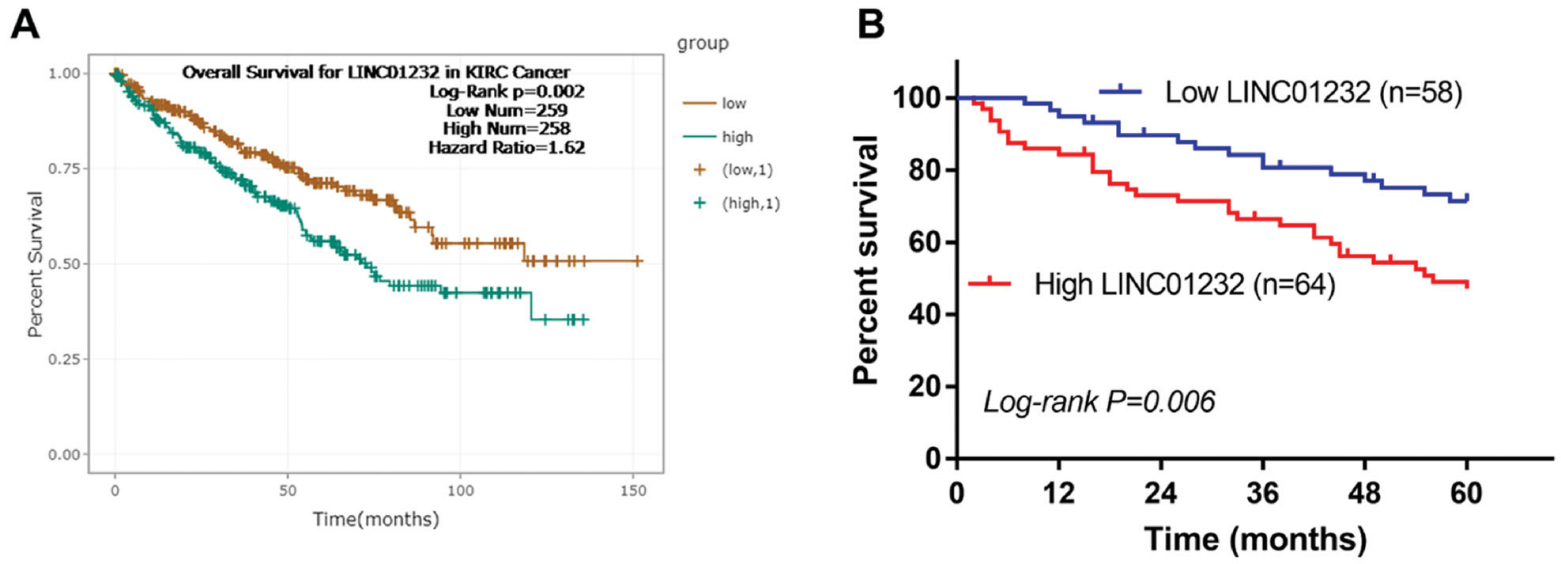
*LINC01232* is associated with overall survival of ccRCC patients. (A) According to the analysis by starBase, high *LINC01232* was associated with poor overall survival of ccRCC patients (log-rank *p* = .002). (B) The results of our study cohort revealed that patients with high *LINC01232* level had short survival time than the patients with low *LINC01232* level (log-rank *p* = .006). LINC: long intergenic non-protein coding RNA; ccRCC: clear cell renal cell carcinoma.

### Association of LINC01232 with the clinicopathological characteristics of ccRCC

In this study, the median value of LINC01232 levels (1.85) was used to divide high and low levels of *LINC01232*. The analysis results of Table 1 showed that *LINC01232* was significantly correlated with tumour size (*p* = .020), lymph node metastasis (*p* = .006) and TNM stage (*p* = .001). In addition, no association was found between *LINC01232* expression and age or gender (all *p* > .05).

**Table 1.** Association of LINC01232 with the clinicopathological characteristics of ccRCC.

Features	No. *n* = 122	LINC01232 expression		*p* Value
		Low (*n* = 58)	High (*n* = 64)	
Age (years)				.845
≤60	41	20	21	
>60	81	38	43	
Gender				.947
Female	48	23	25	
Male	74	35	39	
Tumour size (cm)				.020
≤4	58	34	24	
>4	64	24	40	
Lymph node metastasis				.006
Negative	62	37	25	
Positive	60	21	39	
TNM stage				.001
I–II	62	39	23	
III–IV	60	19	41	

LINC: long intergenic non-protein coding RNA; ccRCC: clear cell renal cell carcinoma.

### High LINC01232 is associated with poor overall survival in ccRCC patients

According to the results of starBase analysis, the overall survival of ccRCC patients with high *LINC01232* levels was significantly worse than that of patients with low *LINC01232* levels (Figure 2(A), log-rank *p* = .002). In our study cohort, we found that high level of *LINC01232* was significantly associated with short survival time, which was consistent with the results of starBase analysis (Figure 2(B), log-rank *p* = .006). Cox regression analysis was performed and the results are shown in Table 2. The results of univariate Cox analysis revealed that lymph node metastasis, TNM stage and *LINC01232* were associated with overall survival in ccRCC patients. The significant variables from the univariate analysis were then included in the multivariate analysis. Multivariate Cox analysis results demonstrated that TNM stage [hazard ratio (HR)=2.084, 95% confidence interval (CI)=1.280–2.841, *p* = .007] and *LINC01232* (HR = 2.207, 95% CI = 1.433–3.086, *p* < .001) were independently correlated with the overall survival of ccRCC patients.

**Table 2.** Cox regression analysis results for patients with ccRCC.

Variables	Univariate analysis		Multivariate analysis	
	HR (95% CI)	*p* Value	HR (95% CI)	*p* Value
Age (>60 years vs. ≤60 years)	1.287 (0.658–1.941)	.667	–	–
Gender (male vs. female)	1.185 (0.812–1.585)	.352	–	–
Tumour size (>4 cm vs. ≤4 cm)	1.485 (0.941–2.007)	.113	–	–
Lymph node metastasis (positive vs. negative)	1.602 (1.089–2.247)	.046	1.396 (0.996–2.097)	.058
TNM stage (III–IV vs. I–II)	2.212 (1.312–3.187)	.003	2.084 (1.280–2.841)	.007
LINC01232 (high vs. low)	2.553 (1.551–3.569)	.001	2.207 (1.433–3.086)	<.001

LINC: long intergenic non-protein coding RNA; ccRCC: clear cell renal cell carcinoma; HR: hazard ratio; CI: confidence interval.

### LINC01232 reduction inhibits ccRCC cell proliferation, migration and invasion

**Figure 3.**
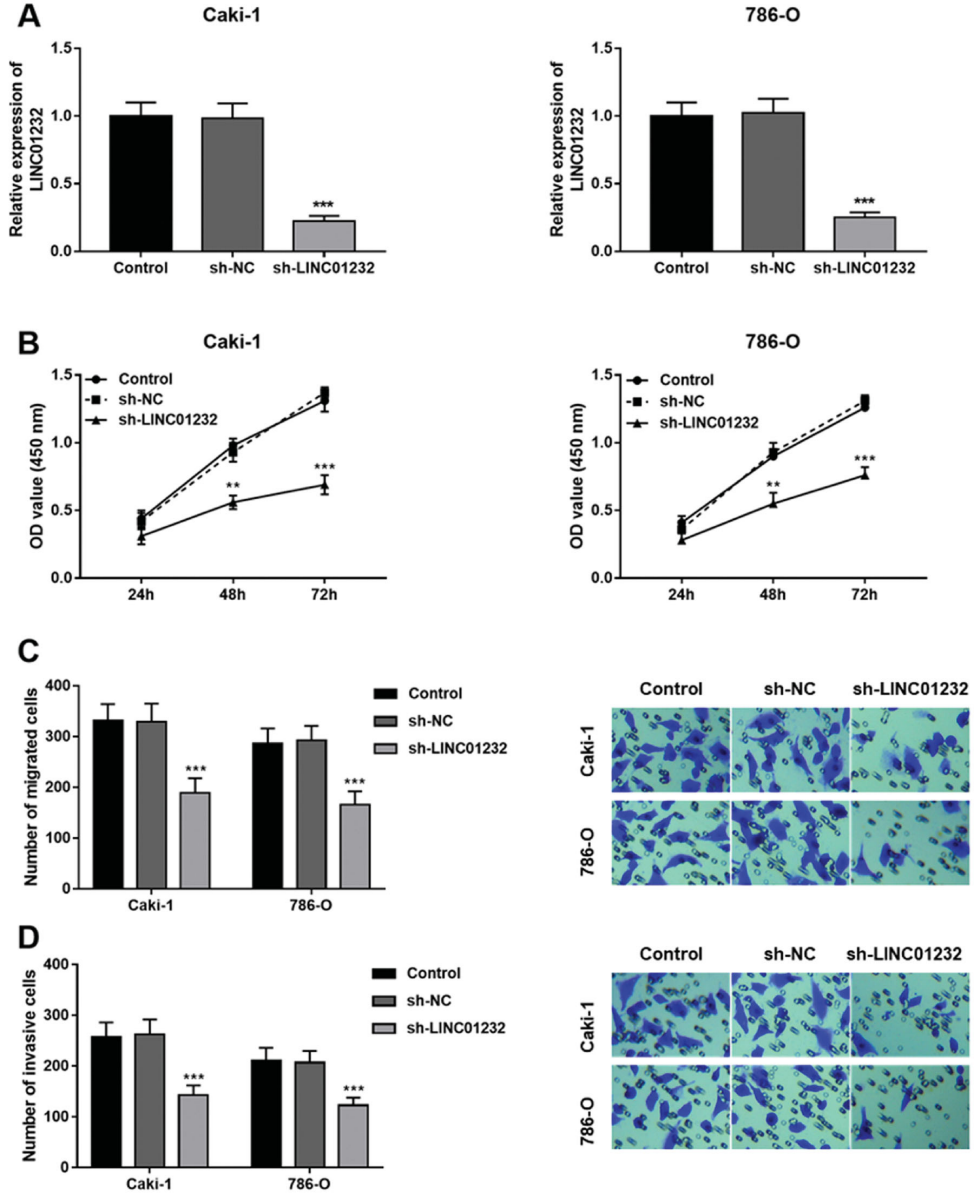
Effects of *LINC01232* on the proliferation, migration and invasion of ccRCC cells. (A) *LINC01232* expression was inhibited by sh-*LINC01232* in Caki-1 (fold change is 0.22) and 786-O cells (fold change is 0.25). (B–D) *LINC01232* silencing inhibited the proliferation, migration and invasion of Caki-1 and 786-O cells. **p < .01, ***p < .001 vs. Control. sh: short hairpin; NC: negative control; LINC: long intergenic non-protein coding RNA; ccRCC: clear cell renal cell carcinoma.

As presented in Figure 3(A), sh-*LINC01232* significantly suppressed the levels of *LINC01232* in the Caki-1 and 786-O cells (all *p* < .001). In addition, the proliferation of Caki-1 and 786-O cells was suppressed by *LINC01232* reduction (Figure 3(B), all *p* < .01). In Caki-1 and 786-O cells, the migration (Figure 3(C), all *p* < .001) and invasion (Figure 3(D), all *p* < .001) were all inhibited by *LINC01232* reduction.

### LINC01232 sponges miR-204-5p in ccRCC

**Figure 4.**
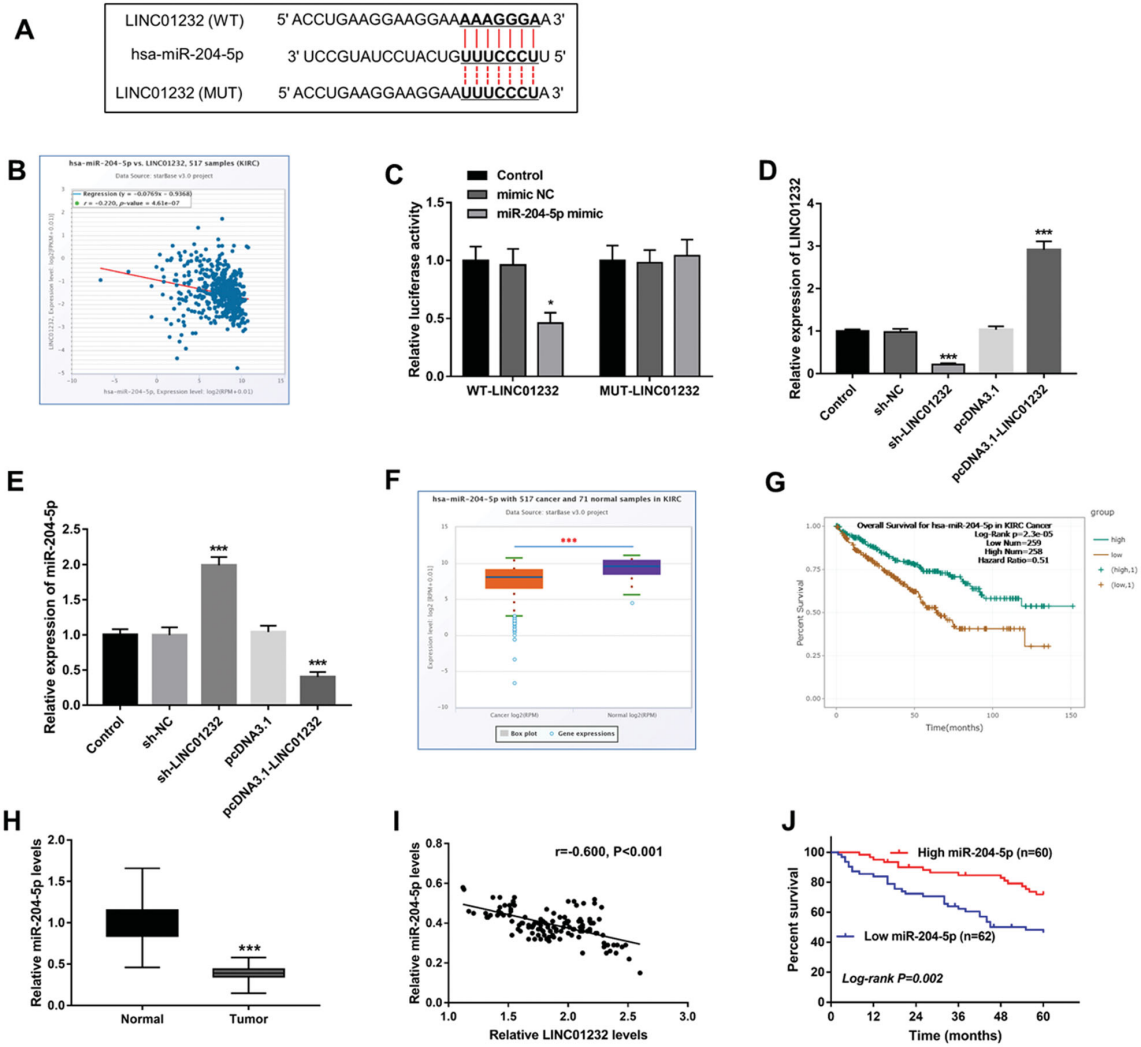
*LINC01232* sponges miR-204-5p in ccRCC. (A) The binding site of *LINC01232* to miR-204-5p was predicted. (B) *LINC01232* was found to be negatively correlated with miR-204-5p in ccRCC by starBase platform (*r*= −0.220, *p* < .001). (C) In Caki-1 cells, relative luciferase activity was significantly inhibited by miR-204-5p upregulation in WT-*LINC01232* group. (D) *LINC01232* expression was downregulated by sh-*LINC01232* and was upregulated by pcDNA3.1-*LINC01232* (fold changes are 0.21 and 2.91). (E) miR-204-5p levels were promoted by *LINC01232* silencing and were suppressed by the overexpression of *LINC01232* in Caki-1 cells (fold changes are 1.97 and 0.4). (F) Through starBase platform, miR-204-5p was significantly downregulated in ccRCC. (G) Through the starBase platform, ccRCC patients with low miR-204-5p levels had a poor overall survival (log-rank *p* < .001). (H) miR-204-5p levels in ccRCC tumour tissues and normal tissues (fold change is 0.39). (I) miR-204-5p levels were negatively correlated with *LINC01232* levels in tumour tissues (*r*= −0.600, *p* < .001). (J) Patients with low miR-204-5p levels had a poor five-year survival (log-rank *p* = .002). *p < .05, ***p < .001 vs. Control or ccRCC tissues from TCGA database or normal tissues from 122 ccRCC patients. WT: wide-type; MUT: mutant-type; sh: short hairpin; NC: negative control; LINC: long intergenic non-protein coding RNA; ccRCC: clear cell renal cell carcinoma.

Through the starBase platform, the binding site of *LINC01232* to miR-204-5p was predicted (Figure 4(A)), and the expression levels of *LINC01232* and miR-204-5p were also found to be significantly negatively correlated in ccRCC (Figure 4(B), *r*= −0.220, *p* < .001). As shown in Figure 4(C), the luciferase reporter assay results showed that the luciferase activity of the WT-*LINC01232* group was significantly inhibited by miR-204-5p overexpression (*p* < .05), and no change was found in the luciferase activity of the MUT-*LINC01232* group. In addition, sh-*LINC01232* inhibited and pcDNA3.1-*LINC01232* promoted relative expression of *LINC01232* (Figure 4(D), all *p* < .001). Moreover, the expression of miR-204-5p was promoted by *LINC01232* silencing and was inhibited by *LINC01232* overexpression in Caki-1 cells (Figure 4(E), all *p* < .001). Analysis of the miR-204-5p expression quantity data in starBase showed that miR-204-5p was significantly lower in ccRCC tissues than that in normal tissues (Figure 4(F), *p*<.001), and the overall survival of ccRCC patients with low miR-204-5p levels was significantly poor (Figure 4(G), log-rank *p* < .001). Analysis of a study of 122 patients with ccRCC similarly demonstrated that miR-204-5p levels were downregulated in ccRCC tumour tissues compared with that in normal tissues (Figure 4(H), *p*<.001) and significantly negatively correlated with *LINC01232* levels in tumour tissues (Figure 4(I), *r*= −0.600, *p* < .001), and that patients with low miR-204-5p levels had significantly lower five-year survival rates (Figure 4(J), log-rank *p* = .002).

### miR-204-5p mediates the regulatory effects of LINC01232 on ccRCC proliferation, migration and invasion

**Figure 5.**
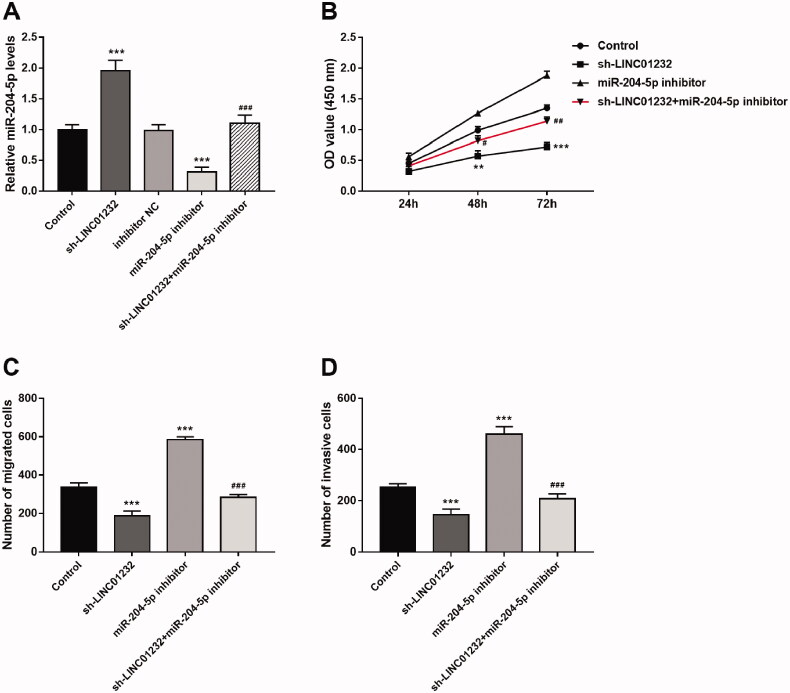
miR-204-5p mediates the effects of *LINC01232* on ccRCC cell proliferation, migration and invasion. (A) The upregulation of miR-204-5p caused by *LINC01232* silencing was reversed by miR-204-5p downregulation in Caki-1 cells (fold changes are 1.96, 0.31 and 1.10, respectively). (B–D) miR-204-5p downregulation reversed the inhibitory effects of *LINC01232* silencing on Caki-1 cell proliferation, migration and invasion. **p < .01, ***p < .001 vs. Control; ^#^*p* < .05, ^##^*p* < .01, ^###^*p* < .001 vs. sh-*LINC01232*. sh: short hairpin; NC: negative control; LINC: long intergenic non-protein coding RNA; ccRCC: clear cell renal cell carcinoma.

As shown in Figure 5(A), miR-204-5p levels were significantly promoted by sh-*LINC01232* and repressed by miR-204-5p inhibitor in Caki-1 cells, and miR-204-5p inhibitor reversed the promoting effect of *sh-LINC01232* on miR-204-5p expression (all *p* < .001). In addition, downregulation of miR-204-5p was found to promote the proliferation, migration and invasion of Caki-1 cells, and miR-204-5p downregulation reversed the inhibitory effects of *LINC01232* silencing on Caki-1 cell proliferation, migration and invasion (Figure 5(B–D), all *p* < .05).

### LINC01232 positively regulates RAB22A through sponging miR-204-3p in ccRCC

**Figure 6.**
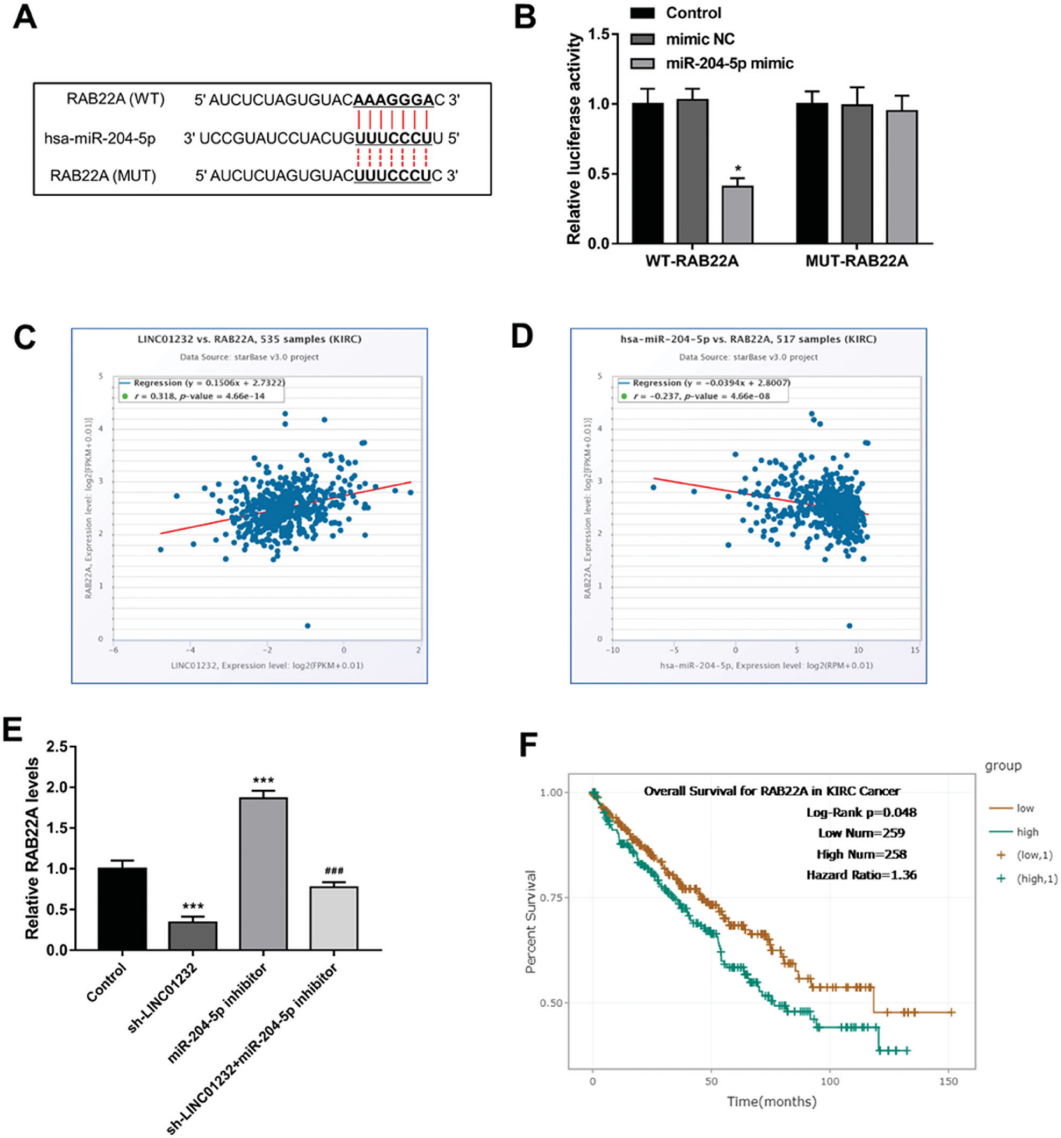
*LINC01232* positively regulates RAB22A through sponging miR-204-3p in ccRCC. (A) The binding sites of miR-204-5p and RAB22A were predicted. (B) miR-204-5p upregulation significantly inhibited relative luciferase activity in the WT-RAB22A group. (C–D) Through the starBase platform, in ccRCC, *LINC01232* was positively correlated with RAB22A (*r* = 0.318, *p* < .001) and miR-204-5p was negatively correlated with RAB22A (*r*= −0.237, *p* < .001). (E) miR-204-5p downregulation reversed the suppressive effects of *LINC01232* silencing on RAB22A levels in Caki-1 cells (fold changes are 0.34, 1.87 and 0.77, respectively). (F) Through the starBase platform, high RAB22A levels were associated with poor overall survival in ccRCC patients (log-rank *p* = .048). *p < .05, ***p < .001 vs. Control; ^###^*p* < .001 vs. sh-*LINC01232*. WT: wide-type; MUT: mutant-type; sh: short hairpin; NC: negative control; LINC: long intergenic non-protein coding RNA; ccRCC: clear cell renal cell carcinoma.

As shown in Figure 6(A), the 3′-UTR region of RAB22A had a binding site for miR-204-5p. Luciferase reporter assay results illustrated that miR-204-5p overexpression markedly inhibited the luciferase activity of the WT-RAB22A group (Figure 6(B), *p*<.05). Analysis of the molecular correlations of the starBase platform demonstrated that *LINC01232* was positively correlated with RAB22A in ccRCC (Figure 6(C), *r* = 0.318, *p* < .001) and miR-204-5p was negatively correlated with RAB22A in ccRCC (Figure 6(D), *r*= −0.237, *p* < .001). As presented in Figure 6(E), silencing of *LINC01232* significantly suppressed, whereas downregulation of miR-204-5p significantly promoted the level of RAB22A in Caki-1 cells (all *p* < .001). Besides, we observed that RAB22A inhibition originally caused by *LINC01232* silencing was relieved by miR-204-5p downregulation in Caki-1 cells (*p* < .001). Through the results of the analysis of TCGA database by starBase platform, we found that high RAB22A level was associated with poor overall survival in ccRCC patients (Figure 6(F), log-rank *p* = .048).

## Discussion

This study is the first to investigate the role of *LINC01232* in ccRCC. Our study demonstrated that *LINC01232* was upregulated in ccRCC tumour tissues and cells and could independently predict the prognosis of ccRCC patients. In addition, it was further found that knockdown of *LINC01232* inhibited the proliferation, migration and invasion of ccRCC cells *in vitro*. Moreover, mechanistic studies indicated that *LINC01232* positively regulated the levels of RAB22A via sponging miR-204-3p to affect ccRCC tumour progression.

Various types of cancer have been found to be affected by lncRNAs [6,15,16]. In addition, some lncRNAs have been revealed to play crucial roles in ccRCC. For example, it has been reported that lncRNA small nucleolar RNA host gene 16 (SNHG16) expression is increased in RCC tissues and ccRCC cells, and the function of ccRCC cells is suppressed by SNHG16 knockdown [17]. Yang et al. have shown that lncRNA homo sapiens HLA complex group (HCG) 18 promotes the development and progression of ccRCC [18]. By using the starBase platform, *LINC01232* showed a significant increase in ccRCC. In our study cohort, we also found a markedly higher level of *LINC01232* in ccRCC tumour tissues and cell lines, and *LINC01232* was markedly correlated with tumour size, lymph node metastasis and TNM stage. Besides, *LINC01232* has been found to be involved in other cancers, such as PAAD [9], pancreatic cancer (PC) [19] and ESCC [11]. PAAD is a type of PC that begins in the ducts of the pancreas. As the most common form of PC, PAAD accounts for approximately 95% of all pancreatic malignancies. Therefore, *LINC01232* may be involved in the progression of ccRCC. Further cellular experimental results demonstrated that *LINC01232* knockdown suppressed the proliferation, migration and invasion of ccRCC cells. In addition, inhibition of *LINC01232* can inhibit ESCC cell proliferation, migration and invasion *in vitro* [11]. Li *et al.* have shown that *LINC01232* regulates cell proliferation and migration in PAAD, suggesting an oncogenic role of *LINC01232* for PAAD [9]. Thus, *LINC01232* may function as a tumour promotor in ccRCC progression and pathogenesis.

Considering the important role of *LINC01232* in ccRCC progression, the clinical significance of *LINC01232* in ccRCC was explored. lncRNAs have been widely reported as prognostic predictors in different types of cancer [20–22]. In addition, some lncRNAs have been reported to be of value for predicting the prognosis of ccRCC, such as lncRNA AGAP2 antisense RNA 1 (AGAP2-AS1) [23] and lncRNA colorectal neoplasia differentially expressed (CRNDE) [24]. The results of the analysis of the TCGA database and our cohort indicated that *LINC01232* was independently associated with survival in ccRCC patients. In addition, *LINC01232* has been demonstrated to serve as an independent prognostic predictor for PAAD patients [10] and PC patients [19]. Moreover, Chen et al. have shown that *LINC01232* levels are associated with shorter overall survival of OC patients [12]. Thus, *LINC01232* may be an independent prognostic biomarker for patients with ccRCC.

Mechanically, an increasing number of studies have shown that lncRNAs can serve as competing endogenous RNAs (ceRNAs) through sponging miRNAs in cancer progression, thereby releasing these miRNAs bound mRNAs from degradation [25]. Some lncRNAs have been found to sponge miRNAs to affect the progression of cancers, including ccRCC [17,18,26]. In addition, *LINC01232* can promote disease progression through sponging miR-654-3p in ESCC [11]. Du et al. have reported that *LINC01232* may sponge miR-370-5p, miR-654-3p and miR-204-5p in PAAD [10]. In this study, we found that *LINC01232* could directly bind to miR-204-5p. *LINC01232* knockdown promoted and *LINC01232* overexpression repressed the level of miR-204-5p in ccRCC cells. Several studies have shown that miR-204-5p is aberrant in ccRCC [27,28] and is involved in ccRCC progression [29]. Consistently, the results of TCGA dataset analysis and our cohort analysis demonstrated that miR-204-5p was decreased in ccRCC tissues and associated with the survival of ccRCC patients, indicating that miR-204-5p may be involved in ccRCC progression. More importantly, miR-204-5p knockdown was found to promote ccRCC cell function and reverse the effect of *LINC01232* on ccRCC cell function. Notably, Wu et al. have reported that lncRNA SNHG4 contributes to RCC progression by sponging miR-204-5p [27]. Therefore, *LINC01232* may play an oncogenic role in ccRCC by sponging miR-204-5p.

RAB22A has been shown to act as a direct functional target of miR-204-5p, the levels of which are regulated by miR-204-5p in several cancers, such as gastric cancer [30] and glioma [31]. In addition, it is noteworthy that a study by Xiong et al. has revealed that miR-204-5p can suppress RCC cell proliferation and invasion by suppressing RAB22A [32]. By starBase platform analysis and our cohort analysis, we found that miR-204-5p could directly bind to RAB22A and negatively regulate the level of RAB22A. In addition, *LINC01232* was found to be positively correlated with RAB22A by starBase platform analysis. Moreover, the repression of RAB22A, caused by *LINC01232* knockdown, was abolished by miR-204-5p downregulation. Furthermore, ccRCC patients with high RAB22A were found to have a poor survival prognosis by the analysis of TCGA database in starBase platform. Thus, RAB22A expression may be associated with the prognosis of ccRCC patients, which will be verified in our future study. The above data indicated that *LINC01232* may regulate the progression of ccRCC by regulating the miR-204-5p/RAB22A axis. Notably, RAB22A was shown to have pro-immunogenic functions in cancer cells [33]. In line with these findings, overexpression of immune-related genes such as PD-L1 was shown to mark high-risk subgroups of ccRCC patients in several cohorts [34], including TCGA ccRCC patients [35]. Given the fact that immune therapy is a crucial part of systemic treatment for metastatic RCC, this information appears relevant and underlines the potential role of the *LINC01232*/miR-204-5p/RAB22A axis in ccRCC.

However, this study has some limitations. First, the study sample size was small, and a large sample should be used for further studies. Second, *in vivo* experiments were not performed in our study, and further investigation of the role of *LINC01232* in ccRCC *in vivo* is warranted. Third, when performing knockdown with shRNA, at least two separate shRNA designs should be used in parallel to study the gene knockdown effects. However, this study only used a shRNA, which is a limitation. Thus, we will use at least two separate shRNA designs for knockdown in future studies. Fourth, miR-204-5p has been found to target other downstream target genes, such as Sirtuin-1 (SIRT1) [36] and runt-related transcription factor 2 (RUNX2) [28], which will be studied in future studies.

In conclusion, this study indicates that *LINC01232*, which is upregulated in ccRCC tissues and cells, may act as an independent prognostic biomarker for ccRCC patients. In addition, *LINC01232* facilitates ccRCC tumour progression via sponging miR-204-5p and increasing RAB22A, suggesting the critical role of the *LINC01232*/miR-204-5p/RAB22A axis in ccRCC progression. Thus, our study may provide potential prognostic biomarker and therapeutic targets for ccRCC.

## Supplementary Material

Supplemental Material

## Data Availability

The data used to support the findings of this study are available from the corresponding author upon reasonable request.
