# Supplementary figures and images for: LINC01232 serves as a novel biomarker and promotes tumour progression by sponging miR-204-5p and upregulating RAB22A in clear cell renal cell carcinoma
Source: Ann Med. 2021 Nov 16;53(1):2153–64. doi: 10.1080/07853890.2021.2001563 (PMC8604453; doi:10.1080/07853890.2021.2001563)

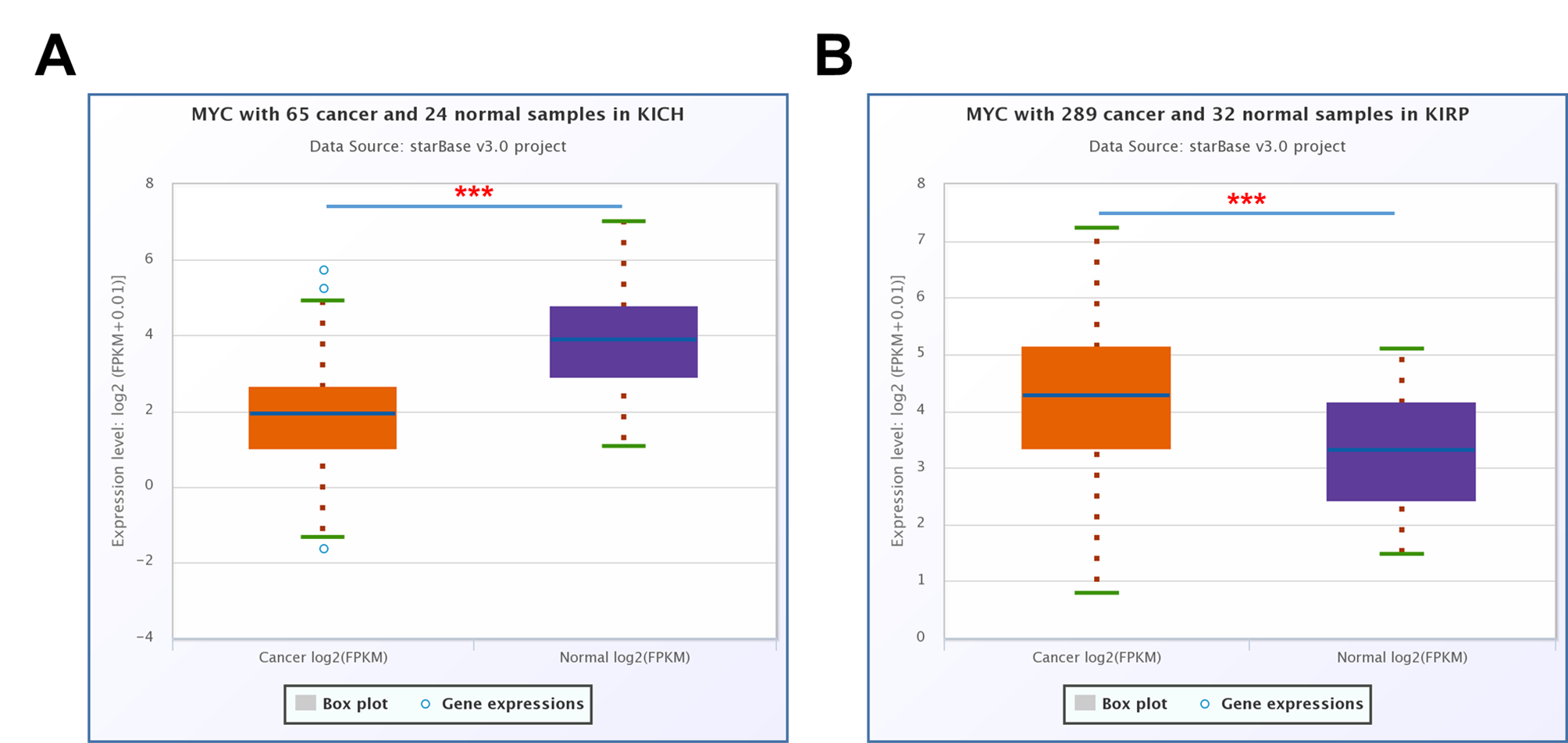

Supplement: Supplemental Material [file IANN_A_2001563_SM8167.zip › Supplemental files/Figure S1.tif]

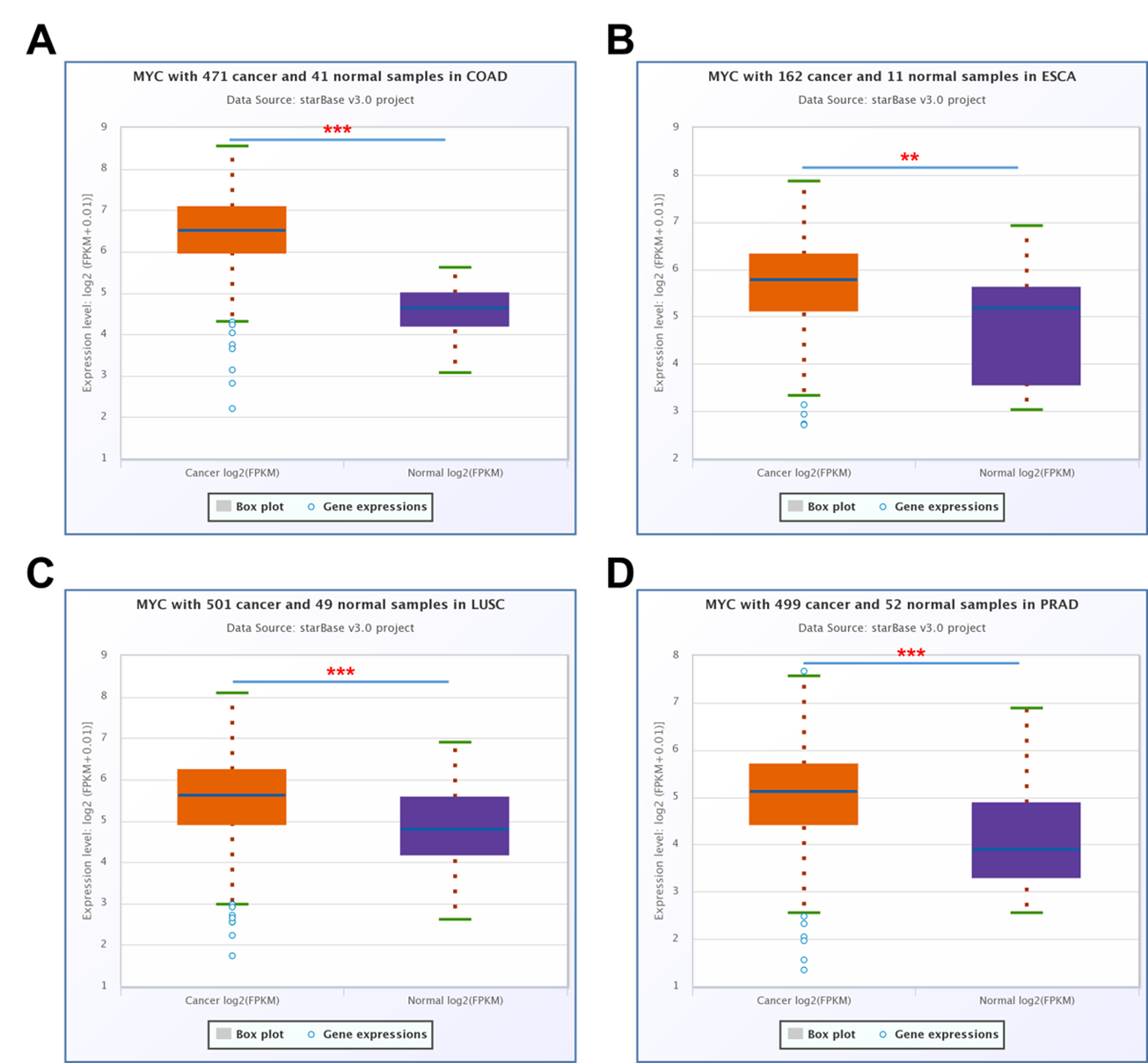

Supplement: Supplemental Material [file IANN_A_2001563_SM8167.zip › Supplemental files/Figure S2.tif]
